# Elderhood and Healthy Aging from an Indigenous Perspective

**DOI:** 10.3390/ijerph22010123

**Published:** 2025-01-18

**Authors:** Yu-Chi Kalesekes Huang, Kathryn L. Braun

**Affiliations:** Thompson School of Social Work & Public Health, University of Hawai‘i at Mānoa, 1960 East-West Road, Biomed D-209, Honolulu, HI 96822, USA; huangyc@hawaii.edu

**Keywords:** aging, indigenous health, elderhood, older adult, community resilience

## Abstract

Researchers have outlined the components of healthy aging, and a 2022 scoping review by Quigley et al. examined healthy aging from Indigenous perspectives. Quigley’s review reinforced the notion that Indigenous health, and thus healthy aging, is a holistic concept. However, no review has specifically addressed Elderhood from an Indigenous perspective. This scoping review aimed to fill that gap by analyzing studies from Quigley’s review and sourcing additional literature on Indigenous Elderhood. Eligible articles identified participants as Indigenous and described Elderhood within the culture. From the 20 included publications, six themes were identified, suggesting that Elderhood was a term limited to adults who were respected for their wisdom, were active in the community, cared for others, passed down Indigenous knowledge, and promoted a vision of the future that built on tradition. Age was not a criterion, as older people who are not seen as respected contributors do not earn the title of Elder. The findings suggest that achieving Elderhood is key to healthy aging for Indigenous adults, regardless of one’s physical health status. Programs and policies offered in Indigenous communities should recognize this distinction. Further research should explore ways to support successful Elderhood, as defined here, as a component of healthy aging in Indigenous communities.

## 1. Introduction

Health disparities between Indigenous Peoples and non-Indigenous Peoples have shown ways that the dominant healthcare systems have failed Indigenous populations. These systems are often deficit-based and often do not consider the importance of historical context and the challenges that Indigenous communities face in today’s society [1]. Indigenous perspectives on health and aging are often excluded by the dominant population [2]. Because understanding of Indigenous Peoples is not thorough, policies and health programs developed by dominant populations may not fit Indigenous needs or worldviews. By undermining traditional knowledge, Indigenous values, and beliefs, services developed for the dominant society and transferred to Indigenous communities can diminish Indigenous autonomy and negatively impact cultural identities. The purpose of this paper is to review Indigenous definitions of Elderhood and explore how these definitions inform notions of healthy or successful aging.

### Definitions of Healthy Aging and Elderhood

In the past few years, considerable effort has been made to define “successful aging”. For instance, Rowe and Kahn’s well-known gerontological model defines three main criteria that must be incorporated for successful aging: (1) relative freedom from disease and disease-related disability, (2) high cognitive and physical functioning, and (3) persistent engagement in life [3,4]. However, other researchers suggest that without consideration of culture, spirituality, resilience and other factors, the definition is not complete [5].

Different ethnic groups have different definitions of health and healthy aging, as well as different worldviews and lifestyles that affect the status of older adults and the operation of family life. Compared with the current mainstream definitions of healthy aging, the definitions ascribed by Indigenous Peoples are different because they have different health experiences and often put more emphasis on place, land, community, and social responsibility to place, land, and community [6]. Indigenous Elders prefer to age in place and maintain strong social connections with community members, which may be based on a definition of healthy aging that differs from the biomedical health promotion approach [7].

A comprehensive review called for programs targeting older adults to broaden their definitions of healthy aging to include elders’ perceived resilience and capacity as older adults [8]. An exploration of perceptions of aging among older First Nations Australians found that mainstream aging policy did not reflect the needs of Indigenous Elders, for whom cultural identity and resilience are important to successful aging [9]. In Indigenous communities, Indigenous Elders’ insights contribute to the reconciliation and resilience of people living and moving forward, and Indigenous Elders are the keepers of life in the community [10]. Most Indigenous Elders feel that intergenerational approaches are essential to promoting health in their communities. For example, research in Alaska found that the definition of healthy aging included strong relations with progeny and community members, with older members as role models who encourage positive relationships across the generations [11].

A recently published review of the literature by Quigley explored Indigenous views of successful aging [6]. The search yielded 31 peer-reviewed publications and a conference report on healthy aging in Indigenous populations. The findings demonstrated that aging well is a holistic concept in these communities, facilitated by spiritual, physical, and mental wellbeing, in which maintaining connections to individuals, place, and culture is crucial [6].

Although findings from these studies suggest that Indigenous groups may have unique definitions of Elderhood as well as healthy aging, a synthesis of knowledge on Indigenous definitions of Elderhood is lacking. Indigenous definitions likely differ from the age-based definitions set by the World Health Organization (WHO) and national governments. Specifically, the WHO sets the threshold for old age at 65 and further categorizes elders into the young old (65–74 years), the advanced old (75–84 years), and the very advanced old (85 years and older) [12]. In the US, Medicare benefits start at age 65, although adults may start participating in some government services at age 60. Because Aboriginal and Torres Strait Islander people in Australia have generally lower life expectancies than non-Indigenous Australians, government reports present information on Indigenous elders at age 50 and older [13]. For similar reasons, the national government of Taiwan defines old age as starting from 65 years generally, but at age 55 years for Indigenous adults [14]. However, other information suggests that age is not a criterion for Elderhood among Indigenous peoples in Australia and Canada [15,16].

The current review aimed to explore definitions of Elderhood within Indigenous communities and consider how these definitions inform notions of healthy or successful aging.

## 2. Methods

Reporting of findings was guided by the Preferred Reporting Items for Systematic Reviews and Meta-Analyses for Scoping Reviews (PRISMA-ScR) [17,18]. Please refer to Supplementary File S1, which contains the PRISMA checklist [19].

### 2.1. Search Strategy

The team of Quigley et al. examined the intricate topic of healthy aging among Indigenous communities, drawing insights from a pool of English-language articles [6]. In contrast, this study took a more focused approach, concentrating specifically on literature that discussed the definition of Indigenous Elders and how this definition intersects with the concept of healthy aging. The primary objective was to determine how the literature identified from the studies identified by Quigley et al., along with their references, addressed the connection between Indigenous notions of Elderhood and perceptions of healthy aging. Thus, the 32 documents included by Quigley et al. were first read in full to see if they addressed the definition of Elderhood in the examined Indigenous community. Then, the references of the articles that mentioned the definition of an Elder were searched for additional studies and government reports. Also consulted was the website of Australian Institute of Aboriginal and Torres Strait Islander Studies.

### 2.2. Inclusion Criteria

The Population, Concept, and Context strategy guided the search and inclusion criteria. The population included Indigenous Peoples. The United Nations recognizes Indigenous Peoples as those groups with historical ties to the lands they live on that maintain distinct social, cultural, economic, and political traits that set them apart from dominant societies and thus face marginalization within their nations. Articles were considered if they identified participants as Indigenous or utilized alternative descriptors such as First Nations, Inuit, Métis, Aboriginal, Māori, Alaska Natives, American Indians, and Native Hawaiian. The Concept was Elderhood and how it was defined in the culture. The Context was Indigenous communities.

### 2.3. Data Screening

The 32 articles from the review of Quigley and the references of these 32 documents were amassed [13]. From this pool, duplicates were removed and the titles and abstracts were reviewed against the inclusion criteria. Following this initial screening, articles of the remaining studies were meticulously examined, and the inclusion criteria were again applied. The remaining articles were included in this review.

### 2.4. Data Review

This review was conducted by two reviewers. The first author is an Indigenous individual from Taiwan. She is a member of the Paiwan tribe and grew up in a tribal family within a Paiwan community in southeastern Taiwan. She also is trained in public health and, in her work, noticed a disconnect between the notions of healthy aging in her tribe and health services in Taiwan that predominantly rely on Western medical frameworks. The second author is a non-Indigenous scholar in gerontology with more than 30 years of experience in research with Native Hawaiian communities in the United States. The second author’s role in this study served to complement the application of decolonial perspectives and to explore the meaning of healthy aging within Indigenous communities.

### 2.5. Data Extraction

The following items were extracted from each study included in the review:

- Full citation.
- Name of Indigenous group and geographic location of the study.
- Study design and approach.
- Quotes related to the definition of Elders and Elderhood.

### 2.6. Data Analysis

Quotes related to the definition of Elder and Elderhood were entered into a spreadsheet, which was reviewed by both authors. The authors discussed each quote and then started to group similar quotes together. Through a continual process of discussing, lumping, and splitting, all quotes were grouped under headers (themes) that the authors felt best described the group of quotes [20]. In the text, themes were reported and illustrated by the most relevant quotes. For tables, documents included in the review were numbered (Table 1 and Table 2) and quotes were summarized (Table 1), with full quotes provided in the text to illustrate the themes.

## 3. Results

### 3.1. Search Results

**Figure 1:**
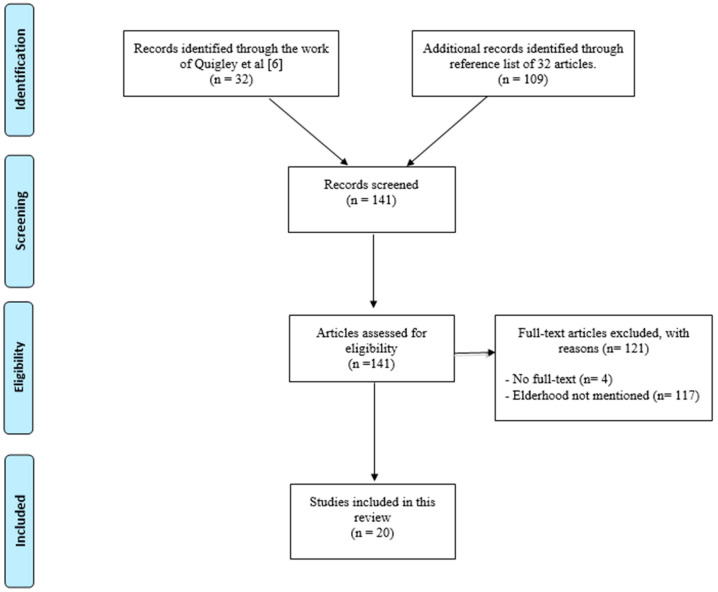
Flow chart [6].

The literature search began with Quigley’s 32 studies and was further expanded by 109 other documents from relevant references cited in these studies. All 141 documents were reviewed in full, and 20 publications met the criteria for inclusion in the review (Figure 1).

Of the 20, eight were from the review of Quigley [6], and the other 12 were identified through citation chasing. Sixteen were peer-reviewed journal articles, one was a conference report, one was a dissertation, and three were government reports from Canada, including governmental reports by the Assembly of First Nations [21], Health Canada [22], and the Royal Commission on Aboriginal Peoples [23]. Two publications were literature reviews, one related to elder abuse [24] and the other related to the role of Indigenous elders in Australia [25]. Ten reported on data collected using qualitative methods [26,27,28,29,30,31,32,33,34,35], and two utilized quantitative methods [36,37]. The three governmental reports [21,22,23] and three other studies [38,39,40] adopted a mixed methods approach.

The Indigenous populations under study showcased a diverse array of cultural backgrounds and geographical locations. In Canada, six studies involved Inuit, Metis, and other First Nation groups. In the US, two focused on Alaska Natives, two on Native Hawaiian populations, one on Samoan and Tongan populations, and one on American Indians. Four studies focused on Aboriginal and Torres Strait Islander communities in Australia, and three on Māori populations in New Zealand. One focused on the Aymara tribe in Chile (Table 1).

**Table 1:** Study number, title, population, design, and definitions.

Study Number	Title	Indigenous Population	Study Design	Definitions of Elderhood
1	Elders and elderlies: Well-being in Indian old age [38]	American Indians, USA	Mixed methods	An Elder is a person of substance and value, a person with inviolate dignity. An Elder is a person held in great respect by his or her community members. Councils of Elders were the active centers of tribal decision-making. Elders held places of prominence in governmental and political affairs. It was through the Elders that the culture of the people was transmitted across generations.
2 Q	Marie’s story of aging well: Toward new perspectives on the experience of aging for Aboriginal seniors in Canada [26]	Métis, Canada	Conference report Case study	Marie demonstrated ongoing contributions to community life. She was valued in this role and could scarcely keep up with invitations to present in classrooms, at workshops, and conferences. Marie was dedicated to the transmission of her accumulated knowledge and wisdom to younger generations.
3	Report of the Royal Commission on Aboriginal Peoples: Perspectives and Realities [23]	Inuit, First Nations and Métis, Canada	Government report Mixed methods	Elders are those recognized and respected for knowing, living, and teaching traditional knowledge. Elders see the world through the eyes of their ancestors and interpret the contemporary world through lessons passed down through generations. Their wisdom is transferred to young people who seek their teachings. Elders are a living bridge between the past and the present. Elders provide a vision for the future, a vision grounded in tradition and informed by the experience of living on the land and safeguarding and disseminating knowledge gained over centuries. Elders have much to contribute in the quest for self-determination and a better relationship among all Canadians. Elders are educators in the broadest sense of the word.
4	Aboriginal elder abuse in Canada [24]	Aboriginals, Canada	Literature review	Not all older people or seniors are deemed to be an elder as defined above and some Elders can be quite young in terms of age.
5	Reaching out: A guide to communicating with Aboriginal seniors [22]	Aboriginals, Canada	Government report Mixed methods	Elder is capitalized when used to indicate honor or a title. It is not capitalized when it is used to mean senior.
6	Sustaining the caregiving circle: First Nations people and aging [21]	First Nations, Canada	Government report Mixed methods	Elders play roles in healing and guidance, and people do not hold preconceived notions of them. Elders have the acknowledgement of their Elder roles by community members. Elders contribute to their communities in a way that is consistent with traditional roles. Elders are perceived to have important wisdom and are respected and valued for their ability to offer guidance to younger generations.
7 Q	If you got everything, it’s good enough”: Perspectives on successful aging in a Canadian Inuit community [27]	Inuit, Canada	Qualitative	Elders, being traditional, are also better people. Elder role models help place pressure on the younger generation to live up to traditional expectations, even in difficult political and economic situations.
8 Q	Away from the islands: Diaspora’s effects on Native Hawaiian elders and families in California [28]	Native Hawaiians USA	Qualitative	Elders have wisdom that comes with age and are willing to share that knowledge. The term kūpuna (Elder) was closely connected to intergenerational learning and caring for mo ‘opuna (grandchildren).
9	Native Hawaiian grandparents: Exploring benefits and challenges in the caregiving experience [29]	Native Hawaiians USA	Qualitative	Native Hawaiian Elders are recognized as major sources of knowledge. Elders play a major role in caring for and raising grandchildren.
10	Cultural context of health and well-being among Samoan and Tongan American Elders [30]	Samoan and Tongan USA	Qualitative	Elders are central to the daunting task of ensuring the transmission of traditional culture. Elders assist young people as they negotiate cross-cultural identities.
11	Te puawaitanga o nga tapuwae kia ora tonu—Life and living in advanced age: A cohort study in New Zealand [36]	Kaumātua (Māori elders), New Zealand	Quantitative	Māori Elders often experience an increase in their roles and responsibilities as they age. Māori Elders are often positioned as the main support of their whānau (family) and hapū (tribe).
12 Q	Taupaenui: Māori positive ageing (Ph.D. dissertation) [31]	Kaumātua, New Zealand	Qualitative	Older Māori have obligations to their whānau that they must balance while attempting to maintain a good life in older age. Older Māori live both in Te Ao Māori, the Māori world, and Te Ao Whanui, the wider society.
13	Oranga Kaumātua: Perceptions of health in older Māori people [39]	Kaumātua, New Zealand	Mixed methods	Māori Elders have an active role in marae (community) activities and support the functions of the marae, hapū (tribe) and whānau. Elders play traditional roles as a resource within their whānau. Elders are often involved with providing care within the whānau.
14	Successful aging in older persons belonging to the Aymara native community: Exploring the protective role of psychosocial resources [37]	Aymara, Chile	Quantitative cross-sectional	Elders play a leading role in cultural transmission. Elders help to maintain Indigenous cultural practices. Elders have high participation in social tasks.
15 Q	Successful aging through the eyes of Alaska Native Elders. What it means to be an elder in Bristol Bay, AK [32]	Alaska Natives, USA	Qualitative	Four elements of Eldership are emotional wellbeing, community engagement, spirituality, and physical health, which are the characteristics of Alaska Natives who have reached Eldership and become respected Elders in their community.
16 Q	Successful aging through the eyes of Alaska Natives: Exploring generational differences among Alaska Natives [33]	Alaska Natives, USA	Qualitative	Elders are role models for young people, who look to them as examples of successful aging. Elders place less emphasis on the biomedical aspects of aging and focus on respecting self and others, remaining active, and making a continuous contribution.
17 Q	Growing old in Kempsey: Aboriginal people talk about their aging needs [34]	Aboriginals, Australia	Private sector report Qualitative	Elders are custodians of knowledge and experience. Elders help the community to continue to develop.
18 Q	Sharing the wisdom of our Elder [40]	Aboriginals and Torres Strait Islanders, Australia	Mixed methods	Elders know that the Sacred Grounds—Connection to Country, Spirituality, and Dreamtime—are all interconnected. Maintaining these connections is critical for older Aboriginal people to live a good life and age well.
19	Older Indigenous Australians: Their integral role in culture and community [25]	Aboriginals, Australia	Literature review	Elder roles include strong kinship relationships, support for the young, and transmission of cultural knowledge. Elders provide a link to Indigenous identity and culture. Elders play important roles in education and childcare. Elders are respected as survivors.
20	Passing on our culture: How older Australians from diverse cultural backgrounds contribute to civil society [35]	Aboriginals, Australia	Qualitative	Elders play an important role in maintaining or promoting their culture. Elders provide support across their communities. Elders help to encourage young people to identify development and to support troubled youth.

### 3.2. Themes

**Figure 2:**
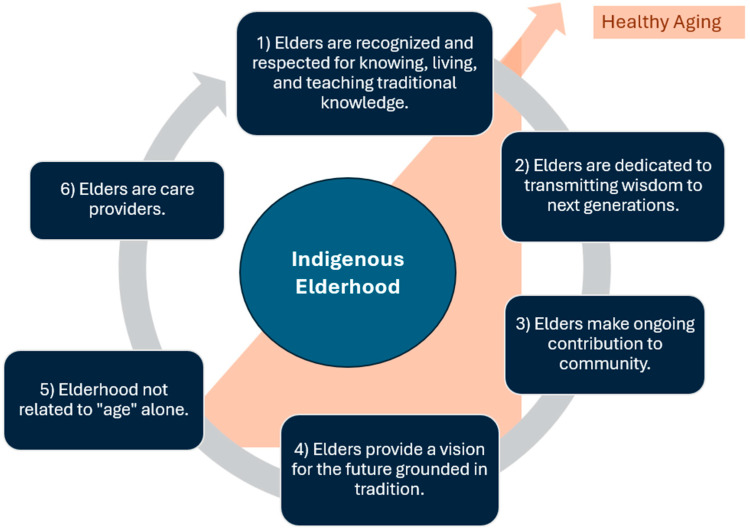
Interrelated definitions of Elderhood.

The analysis identified six major themes, suggesting that an Elder was someone who (1) was recognized and respected for knowing, living, and teaching traditional knowledge, (2) was dedicated to transmitting wisdom to the next generations, (3) made ongoing contributions to the community, (4) provided a vision of the future based in tradition, (5) was not necessarily of a certain age, and (6) was a care provider (Table 2 and Figure 2).

Table 2 presents the six themes from the literature, along with study and citation numbers. It can be observed from this table that the first three themes were mentioned most often (in 14 or more studies), one theme was mentioned in 12 studies, and the other two themes were each mentioned less than nine times each.

**Table 2:** Themes mentioned by study.

	Elders Are Recognized & Respected for Traditional Knowledge	Elders Are Dedicated to Transmitting Wisdom to Next Generation	Elders Make Ongoing Contributions to the Community	Elders Provide a Vision for the Future Grounded in Tradition	Elders Are Not Defined by Age	Elders Are Care Providers
1. Weibel-Orlando [38]	X	X	X	X		
2. Abonyi & Favel [26]		X	X			
3. Report of the Royal Commission on Aboriginal Peoples [23]	X	X	X	X		
4. Dumont-Smith [24]			X		X	
5. Division of Aging and Seniors & Health Canada [22]					X	
6. Assembly of First Nations [21]	X	X		X	X	X
7. Collings [27]	X	X	X	X		
8. Browne & Braun [28]	X	X		X	X	X
9. Mokuau et al. [29]	X	X				X
10. Vakalahi [30]	X	X	X		X	
11. Dyall et al. [36]	X		X			X
12. Edwards [31]	X		X			X
13. Waldon [39]	X	X	X			
14. Gallardo-Peralta & Sánchez-Moreno [37]	X	X		X		
15. Lewis [32]	X	X	X	X		
16. Lewis [33]	X	X	X	X	X	
17. Pearse et al. [34]	X	X	X	X		
18. Delbaere [40]	X	X	X	X		
19. Warburton & Chambers [25]	X	X	X	X	X	X
20. Warburton & McLaughlin [35]	X	X	X	X	X	X
Total	17	15	14	12	8	7

“X” indicates that the reference includes content related to the theme mentioned.

#### 3.2.1. Theme 1. Elders Are Recognized and Respected for Knowing, Living, Teaching Traditional Knowledge

Seventeen of the 20 studies defined Indigenous Elders as those community members recognized and respected for knowing, living, and teaching traditional knowledge. They noted that an Elder is a pillar within their communities, revered for their profound understanding, expression, and transmission of traditional wisdom. Through their lived experiences and intimate engagement with Indigenous traditions, Elders serve as custodians of invaluable teachings, passing down ancestral wisdom to younger generations. As noted by Waldon in his study of the Māori, “Kaumātua [Māori Elders] are crucial to the preservation of our taonga (treasures) for future generations and, most important, we must look after our older Māori, for they are a taonga (asset) for us all [39]” Similarly, Vakalahi found this about Samoan and Tongan Elders [30]:

In relation to the traditional Samoan culture, the key cultural values and practices are taught and enforced by the Elders. These include familial connections; connection to the land; absolute respect for authority of the chiefs (matai) and ministers (faife’au); and a collective identity and worldview that is based on spirituality, physiology, and history.

In their study of the Aymara of Chile, Gallardo-Peralta and Sánchez-Moreno found that [37]:

The significance of the role of older persons in maintaining Indigenous cultural practices is shown in their participation in the social tasks (attendance at community meetings, maintaining the native language, and so on) that represent an essential part of the routine of highland families.

The Report of the Royal Commission on Aboriginal Peoples in Canada [23], which included voices from many Indigenous Elders, noted the importance of Elders as those recognizing and passing on the notion of Indigenous territory:

It is the foundation of everything. Without territory, there is no autonomy. Without territory, there is no home. The reserve is not our home. I am in territory. Language is territory. Belief is territory. It is where I come from. Territory can also vanish in an instant. Before the colonization of the Abitibi, our ancestors always lived on the territory. My grandfather, my grandparents and my father lived there. This is the territory that I am talking about…. (an Algonquin Elder).

#### 3.2.2. Theme 2. Elders Are Dedicated to Transmitting Wisdom to Next Generations

Fifteen of the 20 studies defined Elders as those older adults that showed a profound dedication to the intergenerational transmission of wisdom, serving as vital instruments for the preservation and dissemination of cultural heritage. Their commitment extends far beyond the confines of individual lifespans, as they tirelessly impart valuable teachings, insights, and experiences to the succeeding generations. As quoted by Collings in a study of successful aging in a Canadian Inuit community [27]:

The best thing I like about being Inutquaq is just having the friendship of the other elders of the same generation, the elders around me now. What I enjoy most, too, is being able to talk to young people, people younger than I who have a long life ahead of them, about what life has to offer, what expectations they can have about life, what’s good about life, how they can make that life good for themselves.

This resonates with a quote in Abonyi and Favel about Marie’s story of aging well [26]. Marie is an Aboriginal senior in Canada, who said:

I see that I have moved through the medicine wheel; that my pursuit of more education and experience has taken me through all four quadrants as a teacher (mental and emotional), as a religious educator and sweat leader (spiritual), and in community health education (physical). And having searched the wheel, I found the last piece in health education. And this is where I feel I can make the most contribution to the health and healing of my community. So today I am still involved in many things that are about sharing my life experiences, about helping our youth stay in school, about helping our young people parent well and drawing on the old ways, and about dealing with the hurt that is still there in the high suicide rates among our youth.

Through intimate mentorship, storytelling, and experiential learning, Indigenous Elders nurture the intellectual and spiritual growth of the younger members, guiding them along the pathways of identity formation and cultural belonging. Their teachings encompass a rich tapestry of Indigenous cosmology, traditional ecological knowledge, oral histories, and ceremonial practices, offering profound insights into the connection of all living beings. As Browne & Braun earned in their discussions with Native Hawaiian Elders [28]:

A kupuna (elder) is one who teaches…a kupuna is a respected Elder to learn from, and it’s not about age, but about knowledge and wisdom. Participants suggested that the term kupuna was closely connected to intergenerational learning and caring for mo‘opuna (grandchildren).

#### 3.2.3. Theme 3. Elders Make Ongoing Contributions to Community

Fourteen of the 20 studies suggested that Indigenous Elders demonstrate ongoing contributions to community. Elders hold respected positions of leadership and authority within Indigenous communities, where their guidance is sought in matters of governance, policymaking, and community development. Their perspectives, grounded in traditional values and community priorities, contribute to informed decision-making processes that reflect the collective interests and aspirations of the community. Quantitative research by Weibel-Orlando found that Elderhood involved active involvement in Indian community life, regular interaction with family (particularly grandchildren), continued community contribution and service, personal acts of altruism, and community recognition of such good works [38]. In another example, Dyall et al. found that “the role of kaumātua/kuia (Māori Elders) is very demanding on one’s time and health: long hours spent at tangihaga (funeral ceremonies) and marae (community) meetings [36]”. In Vakalahi [30], a Samoan Elder said,

Our self-esteem and self-worth increase when we give or help others. The more we give, the happier we are so when we accumulate wealth (foods, animals, land and crafts), it is to give and donate to others when needed, for one day I may need help myself.

In their study of Aboriginal Australians, Warburton and McLaughlin heard this example of contributing to others: “I suppose in a nutshell we’re extended families, so no matter who you are, what you are, where you come from, whether relations or non-relationships [35]. Just treat everyone as extended family if you can help… you’re always there to help”.

Lewis found that the community is a focal point and serves an important role in the Elders’ lives [32]. Community engagement provides the Elders with a sense of purpose and a role in the community. In turn, the community cares for older adults, as in these two examples: “We get company. Like, when you are sick, they’ll help you. They don’t leave you to be by yourself” and “I don’t see people not being sent home [vs to a facility]. Family support systems are there”.

#### 3.2.4. Theme 4. Elders Provide a Vision for a Future Grounded in Tradition

Twelve of the 20 articles defined Indigenous Elders as those older adults who serve as visionary leaders that bridge the past, present, and future. As noted in a report from the Assembly of First Nations in Canada, “the term Elder rather than senior celebrates the vitality, knowledge, experience and positive contribution of our Nations’ Elders to our common future” [21].

Grounded in the wisdom of their ancestors, Elders possess a unique ability to envision a future that is harmonious with the natural world and aligned with the principles of intergenerational equity and community well-being. As noted from the study of Warburton and Chambers of Aboriginal Australians [25]:

We are the women who are fighting to keep the culture going. We’ve been teaching the younger women and the women that were taken away, teaching the people the lost culture. We really know the land. We were born on the Manta (land), born on the Earth. And never mind our country is in the desert, that’s where we belong, in the beautiful desert country. The learning isn’t written on paper as whitefellas’ knowledge is. We carry it instead in our heads and we’re talking from our hearts, for the land.

Central to their role is the cultivation of a forward-looking mindset that acknowledges the connection of all aspects of life, including physical, mental, emotional, and spiritual health. As heard by Browne and Braun in their study of Native Hawaiians [28]:

How do I think like a Hawaiian? I see the people I work with, and I think I’m more patient than they are—we are a more giving people, more family oriented. Our children and family are most important… To us the land—we are in tune with sky and land. More with nature, I think.

The report of the Assembly of First Nations from Canada concludes that the Indigenous perspective on the life course, as conveyed by Indigenous Elders, is characterized by a cyclical rather than linear understanding [21]. Additionally, it encompasses the belief in the continuity of life beyond death, wherein existence persists in the spiritual realm and through one’s progeny.

Moreover, Indigenous Elders play a crucial role in guiding community efforts to address contemporary health challenges, drawing upon traditional healing modalities and Indigenous ways of knowing to promote wellness and prevent illness. Their visioning for the future is a critical and demanding responsibility, as heard by Waldon in his study of Māori Elders, “As guardians of te reo Māori (language), ngā tikanga (law) and nga iwi (tribe), Hapū (tribe), and Whānau (family), kuia and kaumātua (respected Elders) have demands placed upon them which have no equivalent in Pākehā (White) society”.

#### 3.2.5. Theme 5. Elderhood Is Not Related to “Age”

Eight of the 20 studies specified that, in Indigenous cultures, the concept of Indigenous Elderhood is not defined by age. For example, in his study of Alaska Natives, Lewis heard, “I don’t think it is just age—you don’t determine if you are an Elder, the community does [33]” and “some of us merely become elderly, but don’t become an Elder”.

As similar sentiment was noted in the report of the Assembly of First Nations from Canada [21].

It is important to acknowledge the distinction between the terms “Elder” and “older person” within an Indigenous context. “Elder” refers to individuals who hold distinguished roles within their community and serve as guardians of cultural heritage. In contrast, “older person” is a broader term used to describe individuals above a certain age, typically around 50 years old.

Thus, being an Indigenous Elder transcends chronological age, and rather encompasses a deep-seated respect for wisdom, experience, and cultural knowledge. While chronological age may be a factor, it is not the sole determinant of Elderhood; rather, it is the embodiment of traditional teachings, values, and leadership qualities that distinguish individuals as Elders within their communities. In many Indigenous societies, Elders are sought after for their guidance and spiritual insight, drawing upon a lifetime of experiences and intimate connections with the land, ancestors, and community members. as heard by Collings in his study in Canada [27].

Elders doing good are always keeping busy with their life. I always see them in the community doing completely different things. I see them in one place doing one thing and then an hour, two hours later I see them somewhere else doing something else. I wonder where they get the energy from.

Interestingly, engaged Indigenous Elders believe they have better health, as noticed in work with Aboriginal Australians of Delbaere [40].

… I’m more like—42… I certainly don’t feel 72. And I don’t behave like it… I’m still thinking about [joining] rock-and-roll bands… So, the age thing, I guess it must literally be something that’s individual, to a certain degree. Because I know people twenty years younger than me who have given up.

#### 3.2.6. Theme 6. Elders Are Care Providers

Seven of the 20 studies noted that important role of Indigenous Elders in providing care within the family. For example, Waldon surveyed older Māori and found that “90% reported caring for children, while 80% cared for sick whānau members” [39]. As found by Mokuau et al., the role of caregiver extends far beyond mere physical assistance [29]; it encompasses emotional, spiritual, and cultural care that addresses the well-being of the whole person.

I taught them to be humble, respectful, to say hi to everyone and kiss them hello [in the Hawaiian way]”. The value of ‘ohana (family) also was transmitted… You have to take care of one another, love each other, help each other out. That’s why you have ‘ohana, to help each other out.

Intergenerational care is expected as it was received. And this caring included the transmission of heritage, culture, and lessons through stories, songs, and teachings that have been handed down through the ages. For example, Warburton and McLaughlin heard from an Indigenous Australian Elder [35]:

My Grandmother lived with us, and she taught us all the old ways and told us all the old Koorie (creation) stories. They’d always tell us kids all the stories when we were goin’ to sleep. If your mother didn’t tell you stories, then your grandmother did. All the kids would fight over who slept with Gran, she was such a good storyteller.

The study of Aboriginal Australians by Pearse noted this about intergenerational caregiving [34]:

It was clear that the relationship with their grannies was extremely important to the participants. Most participants said that these relationships add meaning to their lives and that it is these relationships that keep them going. Although it was clear that raising grandchildren puts a lot of pressure on older people, this was also seen by participants as a joy and strength. Some said that it makes life worth living.

## 4. Discussion

While Quigley et al. provided a comprehensive view of Indigenous perspectives on healthy aging, this study focused on the definition of Elderhood in Indigenous communities [6]. Six themes were identified in this study, suggesting that Indigenous Elderhood is not defined by age alone or physical health status, but rather by an older person’s commitment to preserving and transmitting Indigenous culture, continuing to contribute to family and community, and providing a vision for the future based on tradition. By demonstrating these behaviors, an older person can be called an Elder and therefore deemed to exhibit healthy aging. These findings correspond with the research by Yashadhana et al., who found that conventional aging models fail to adequately encompass the needs and values of older First Nations Australians [9]. It also resonates with Lewis’s research on the concept of generativity in late among Alaska Natives, which suggests that Elders’ contributions and cultural transmission are closely related to healthy aging [41].

The identified themes are clearly interconnected and overlapping. For example, those who are recognized and respected for knowing, living, and teaching culture (Theme 1) may also be providing care, especially to grandchildren (Theme 6), and this provides them the opportunity to transmit culture to the next generation (Theme 2). Those who are recognized and respected for knowing, living, and teaching culture (Theme 1) also contribute to the community (Theme 3) and can provide a vision for the future based on tradition (Theme 4). Thus, Figure 2 shows the themes in a circle.

Findings highlight considerations for the development of culturally sensitive aged and healthcare services. For example, Indigenous Elders should inform the design and delivery of health education and outreach. Recognizing the value Elders can play in these roles would support their community’s health while providing Elder’s motivation to maintain good health as they safeguard the cultural vitality of their entire family and community. Numerous studies have highlighted the significant influence that Indigenous Elders can play in promoting community health [42,43,44,45,46,47].

The findings of this study also suggest that recognizing and supporting the role of Elders in the community will not only contribute to achieving healthy aging for themselves and promoting health in their communities, but it may also enhance community resilience. In the context of rapid cultural erosion, Indigenous Elders, as bearers of cultural knowledge, are necessary to preserving traditional culture and knowledge. As shown in the research by Usher, Aboriginal community resilience has emerged as a response to the collective experience of adversity, including transgenerational grief and loss. Resilience is supported through the development and maintenance of support structures and shared resources through cultural practices, which in turn strengthen bonds and promote mutual reciprocity. Such dynamics enable communities to engage in transformative strategies to effectively address challenges [48]. These findings also reinforce the work of Ballard et al. that Indigenous Elders could be roles models for decision-making and providing recommendations towards reconciliation for Indigenous peoples in Canada [10].

The cultural identity of Elders and their resilience in both traditional and modern societies should be considered in policies for healthy aging. In a society characterized by multicultural integration, it is imperative for governments to acknowledge the values of each ethnic group, particularly in the context of Indigenous populations. Due to often limited sample sizes, data from studies with Indigenous populations may be deemed to lack research value. This thinking reflects the ongoing harm inflicted by colonial policies on Indigenous communities and Elders. More researchers and policymakers must stop discounting Indigenous beliefs and research on these beliefs and take them into account as policies and programs are developed and evaluated. An initial grasp of the definition of Indigenous Elderhood and its significance to the overall health of the community is a start.

The objective of this scoping review was to examine the definitions of Elderhood within Indigenous communities and to elucidate how these definitions inform healthy aging. Such inquiry contributes to enhancing both the depth and breadth of academic research in this area. Continued research is needed, however, to enhance our understanding and awareness of the roles of Elders within tribal contexts. Scholars should be encouraged to adopt methods such as community-based participatory research and participatory action research [49]. These approaches allow Indigenous peoples to take the lead in research and exploration, facilitating the integration of their traditional values into policy recommendations and program design.

### Strengths and Limitations

This review is subject to limitations. It exclusively included articles published in English, which may have resulted in the omission of studies involving Indigenous Peoples from non-English speaking countries, including those in Africa, Asia, and Europe. A thorough search of the web was not conducted, nor were studies assessed for scientific rigor.

The studies included in this research reflect themes defined by Indigenous Elders from various communities. The definition of Eldership may vary across different Indigenous communities and nations, with differences across and even within individual communities. Rather than suggest a universal definition of Indigenous Elderhood, this study synthesized research conducted at a specific point in time, with the hope that future studies can build upon this research. Qualitative research could be conducted through which themes are shared with specific Indigenous groups to validate, contradict, and extend findings from this review. For example, in research by the first author with older adults in the Indigenous Paiwan tribe in southwest Taiwan, participants characterized an Elder who exemplifies healthy aging as one who practices and transmits cultural traditions, engages in lifelong learning, and contributes to their family and tribe, regardless of physical health status [50].

## 5. Conclusions

In conclusion, Indigenous definitions of Elderhood are not age-based, nor do they conform to the Western conceptualization of healthy aging as a result of maintaining functional ability [51], or the notion that healthy aging is within reach of anyone who has good physical and cognitive health and stays engaged. In contrast, Indigenous communities believe that an older person must demonstrate commitment to the culture, community, and next generation before being deemed an Elder, and once they are an Elder, they can be said to have achieved healthy aging. The six interconnected themes identified here emphasize the holistic and indispensable role of Indigenous Elders in fostering cultural resilience, intergenerational continuity, and communal harmony. To become an Elder means to embrace this role, and this leads to healthy aging.

## Data Availability

Articles and extraction tables are available from Y.-C.K.H.

## Supplementary Materials

The following supporting information can be downloaded at: https://www.mdpi.com/article/10.3390/ijerph22010123/s1, File S1: Preferred Reporting Items for Systematic Reviews and Meta-Analyses extension for Scoping Reviews (PRISMA-ScR) Checklist.
